# Supplementary material for: Alkaline-based aqueous sodium-ion batteries for large-scale energy storage
Source: Nat Commun. 2024 Jan 17;15:575. doi: 10.1038/s41467-024-44855-6 (PMC10794691; doi:10.1038/s41467-024-44855-6)
Supplement: Supplementary file 1 — Supplementary Information [file 41467_2024_44855_MOESM1_ESM.pdf]

Supporting information for

Alkaline-based aqueous sodium-ion batteries for large-scale energy storage

Han Wu[1,†], Junnan Hao[1,†], Yunling Jiang[1], Yiran Jiao[1], Jiahao Liu[1], Xin Xu[1], Kenneth Davey[1], Chunsheng Wang[2] & Shi-Zhang Qiao[1,*]

[1] School of Chemical Engineering, The University of Adelaide, SA 5005, Australia

[2] Department of Chemical and Biomolecular Engineering, University of Maryland, College Park, MD 20742, USA

[†] These authors contributed equally to this work: Han Wu, Junnan Hao

* Corresponding author's email: s.qiao@adelaide.edu.au

Supplementary Discussion

Text S1. Function of carbon in Ni/C coating.

The binder was Nafion-Na which forms a dense coating on the surface of the cathode. With low ionic conductivity, it increases interfacial resistance and compromises rate performance. Adding carbon matrix improves the porosity of the polymer and conductivity of the coating, ameliorating the detrimental effect of the coating on rate performance. As shown in **Supplementary Figs. 13a-b**, following the introduction of the carbon matrix, the resistance of the coating with a thickness of 25 μm, was reduced from *ca*. 150 to < 10 Ω, evidencing that carbon addition reduces the interfacial resistance of the coating. However, because of the absence of Ni, the carbon coating itself cannot induce a H_3O^+-rich local environment to suppress OER and cathode dissolution. Therefore, batteries with pure carbon coating exhibit significant over-charging caused by water decomposition (**Supplementary Fig. 13c**) and poor cycling performance induced by cathode deterioration (**Supplementary Fig. 13d**).

Text S2. The barrier effect of coating on OH^- migration.

Experiments were conducted to establish the barrier effect of this coating material on OH^- migration. An H-cell configuration, illustrated in **Supplementary Fig. 16**, was used. An alkaline electrolyte with a pH of 12.3 was introduced into the left-side of the H-cell, whilst a neutral electrolyte with a pH of 6.5 was added to the right-side, in which the uncoated cellulose, Nafion-Na coated cellulose, and Ni/C coated cellulose were used as separators, respectively. During the resting phase, OH^- ions in the left-side of the H-cell migrate to the right-side, resulting in an increase in pH in the right-side. To quantify this effect, a pH meter was used to determine the rate of pH increase in the right-side. As seen in **Supplementary Fig. 17**, in comparison with the uncoated cellulose, the Nafion-Na coated cellulose exhibits a suppression of OH^- ion migration, and the Ni/C boosts this effect.

Text S3. Transformation of Ni in coating and contribution to H_3O^+-rich local environment. Generation of H_3O^+ is predicated on two transformation steps, 1) irreversible oxidation of Ni → $Ni(OH)_2$, which consumes OH^- anions on the cathode surface, and 2) reversible $Ni(OH)_2$ ⇌ NiOOH. The irreversible step was confirmed by the 'gradually' decreasing peak intensity for Ni (111) and growing intensity for peak intensity related to $Ni(OH)_2$ in XRPD patterns during battery charging (**Supplementary Figs. 15a-b, Eqn. S1**). However, following charging to > 1.8 V, the peak exhibiting $Ni(OH)_2$ vanished and a new peak related to NiOOH appeared, evidencing the transformation of Ni^{2+} to Ni^{3+} (**Supplementary Figs. 15b-c**). Additionally, this reaction generates H^+ to initiate a H_3O^+-rich local environment (**Eqn. S2**). NiOOH can be reduced to $Ni(OH)_2$ following discharging where it is beneficial to dynamically maintain the H_3O^+-rich local environment. As seen in **Supplementary Fig. 18**, the specific capacity for Ni/C is *ca*. 150 mAh g^{-1}. The Ni in the coating is < 0.1 mg cm^{-2} and the electrode loading is *ca*. 10 mg cm^{-2}. Therefore the capacity provided by the Ni/C is < 1.5 mAh g^{-1}.

$$Ni + 2OH^- \rightarrow Ni(OH)_2 \quad \text{(S1, during charging, irreversible)}$$

$$Ni(OH)_2 \rightleftharpoons NiOOH + H^+ \quad \text{(S2, reversible)}$$

The oxidation of Ni and reversible transformation between $Ni(OH)_2$ and NiOOH were confirmed *via* soft XAS. As shown in **Supplementary Fig. 19**, Ni has the greater spectral mass in two locations in the L_3 edge absorption band (peak A in the lower, and peak B in the higher energy region). The intensity ratio peak B/peak A is good evidence for state changes in Ni element[1,2]. An increase in the intensity ratio evidences the oxidation of Ni, whilst a decrease evidences the reduction of Ni. In the pristine state, the average oxidation state for Ni will be close to Ni^0. Following charging to 2.2 V, the ratio peak B/peak A increases, confirming the oxidation of Ni. However, following discharging to 0.5 V, the intensity ratio decreases, evidencing that Ni oxidation and reduction are partially reversible during battery cycling.

Supplementary Figures

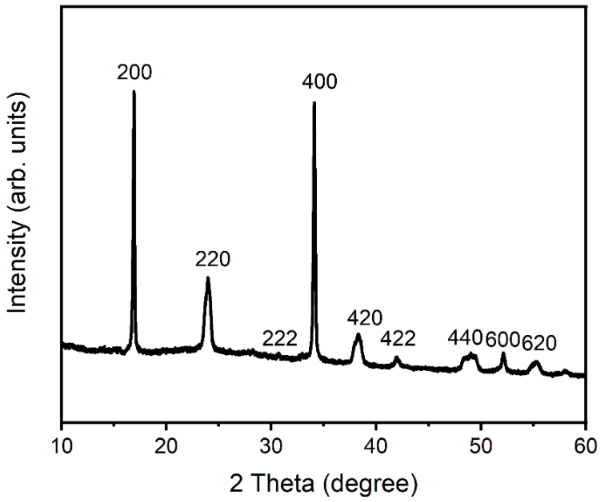

Supplementary Fig. 1 | X-ray diffraction (XRD) pattern for $Na_2MnFe(CN)_6$ (NMF) cathode. The pattern confirms crystallinity for NMF in good agreement with reported findings[3].

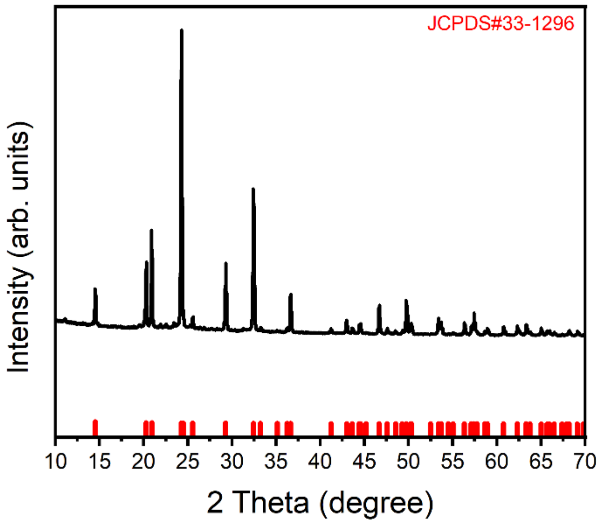

Supplementary Fig. 2 | XRD pattern for $NaTi_2(PO_4)_3$ (NTP) anode. The pattern for synthesized NTP anode agrees well with the Powder Diffraction File (PDF) standard card for $NaTi_2(PO_4)_3$.

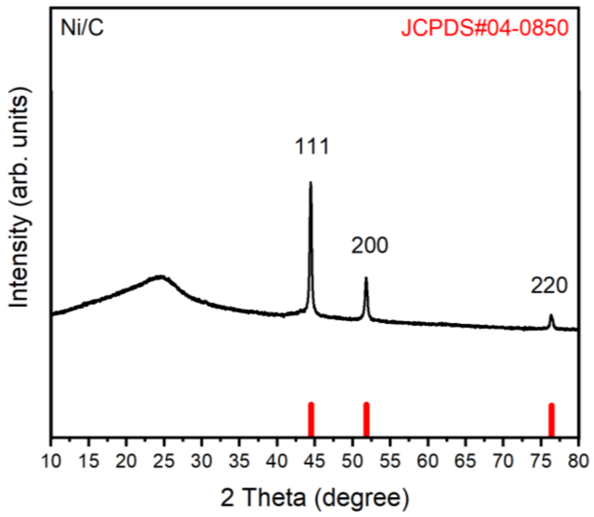

Supplementary Fig. 3 | XRD pattern for Ni/C. The pattern confirms hybrid of Ni/C.

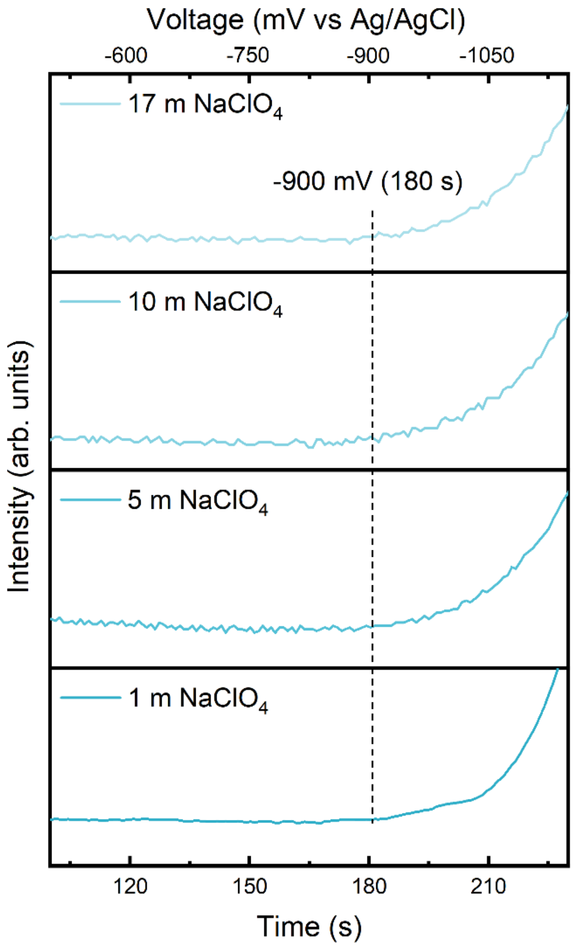

Supplementary Fig. 4 | *In-situ* DEMS tests for $NaClO_4$ electrolytes at a rate of 5 mV s^{-1}. Au-coated PTFE as the working electrolyte, Pt-wire as the counter electrode and Ag/AgCl as the reference electrode.

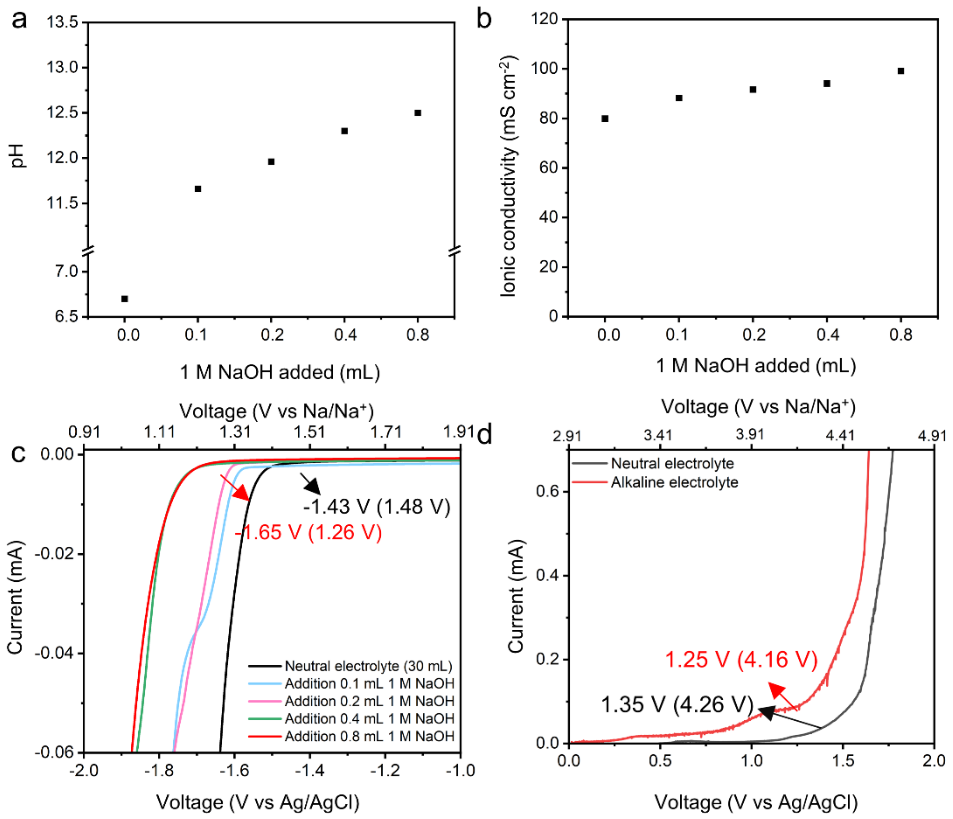

Supplementary Fig. 5 | Electrolyte characterization using three-electrode system (with glass carbon as the working electrode, Ti as the counter electrode, and Ag/AgCl as the reference electrode). **a,** pH for neutral electrolyte following the addition of selected volumes of 1 M NaOH. **b**, Ionic conductivity for neutral electrolytes following the addition of selected volumes of 1 M NaOH. **c**, LSV curves for electrolytes with NaOH addition from −1.0 to −2.0 V. **d,** LSV curves for different electrolytes from 0 to 2.0 V.

As evidenced in **Supplementary Figs. 5a-b**, with the addition of 1 M NaOH (from 0 to 0.8 mL), the pH and ionic conductivity for the electrolyte (30 mL) increase, which lowers cathodic limiting potential (**Supplementary Fig. 5c**). From LSV curves in **Supplementary Fig. 5c**, the electrolyte with 0.4 mL NaOH addition exhibits similar HER overpotential to that with 0.8 mL NaOH addition. The alkaline system with 0.4 mL NaOH addition was therefore selected as an example. However, the increased alkalinity with 0.4 mL 1 M NaOH addition boosted OER at the cathode (**Supplementary Fig. 5d**).

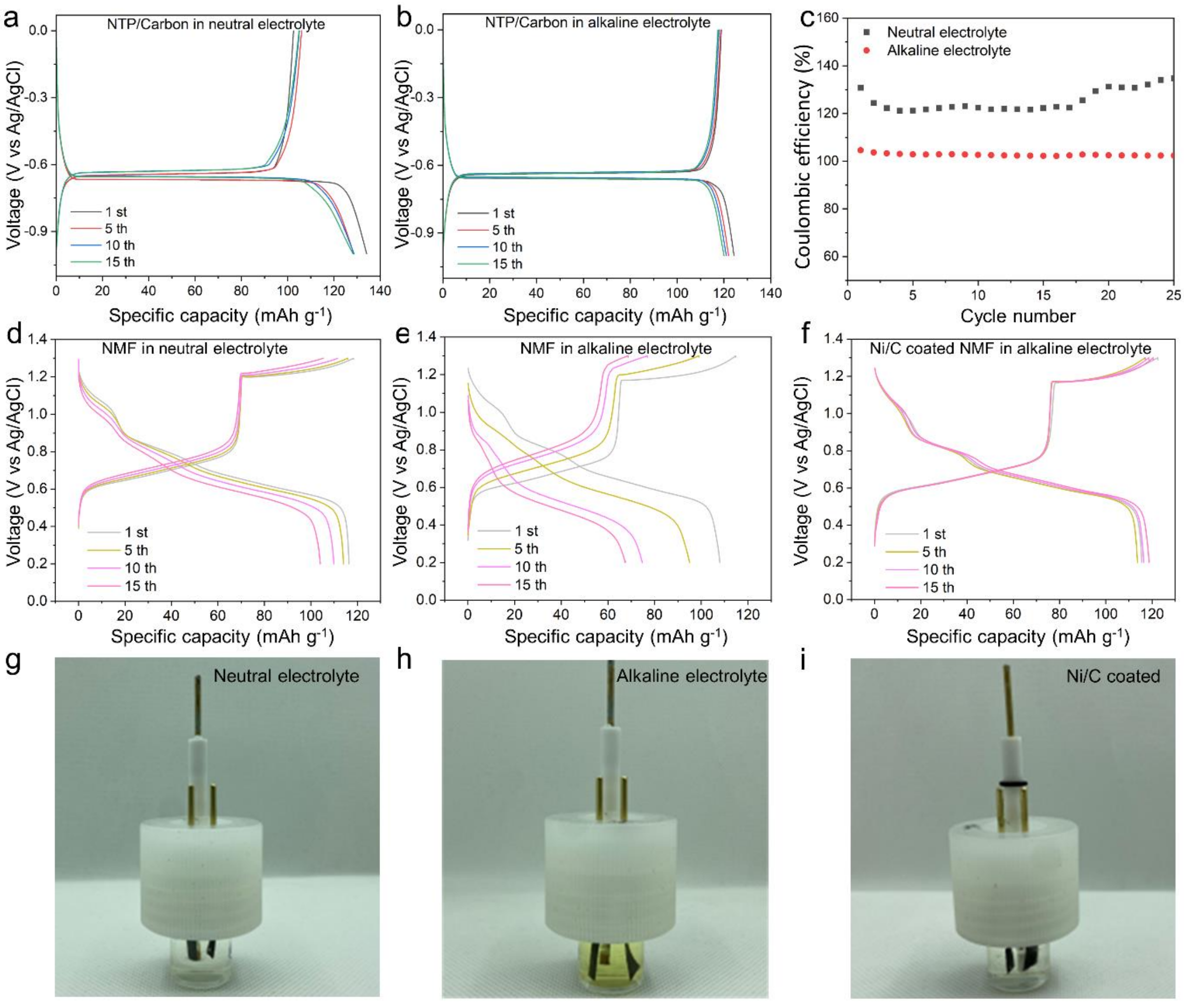

Supplementary Fig. 6 | Cycling performance for half-cell using three-electrode system (Ag/AgCl electrode with sat. aq. KCl solution as reference electrode and carbon black as counter electrode). Charge and discharge profiles for NTP electrode in a three-electrode cell using **a**, Neutral and **b,** Alkaline electrolyte. **c**, Coulombic efficiency (CE) comparison for NTP electrode in a three-electrode cell using neutral and alkaline electrolyte. Charge and discharge profiles for NMF electrode in **d,** Neutral and **e,** Alkaline, electrolyte and **f,** Alkaline electrolyte with Ni/C coating. Digital photograph of cycled NMF electrode in a three-electrode system using **g,** Neutral and **h,** Alkaline, electrolyte and **i,** Alkaline electrolyte (NMF with Ni/C coating).

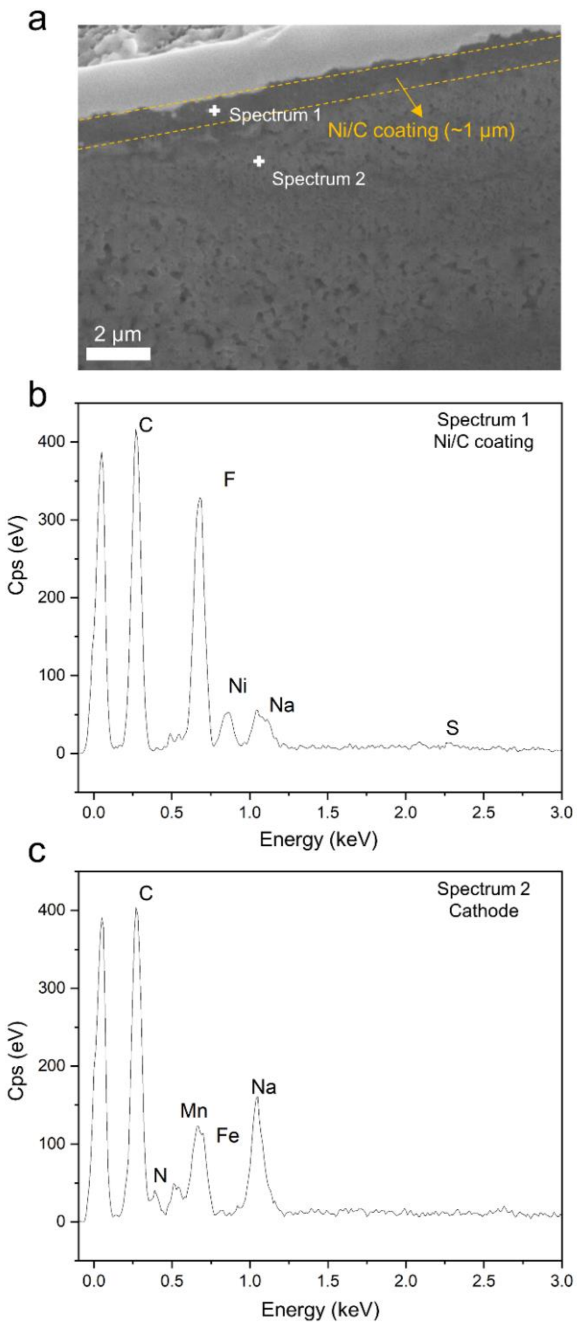

Supplementary Fig. 7 | Interface structure characterization of coated cathode. a, Cross-section SEM image of Ni/C coated NMF. **b-c,** EDS point scan for cross-section SEM image.

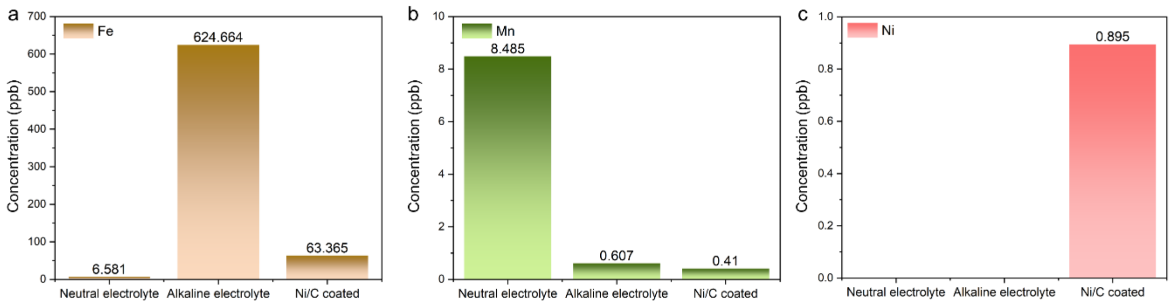

Supplementary Fig. 8 | ICP-MS findings for electrolytes collected from three-electrode cells after cycling with/without Ni/C coating on the cathode surface in neutral/alkaline electrolyte. a, Fe, **b**, Mn and **c**, Ni, concentration.

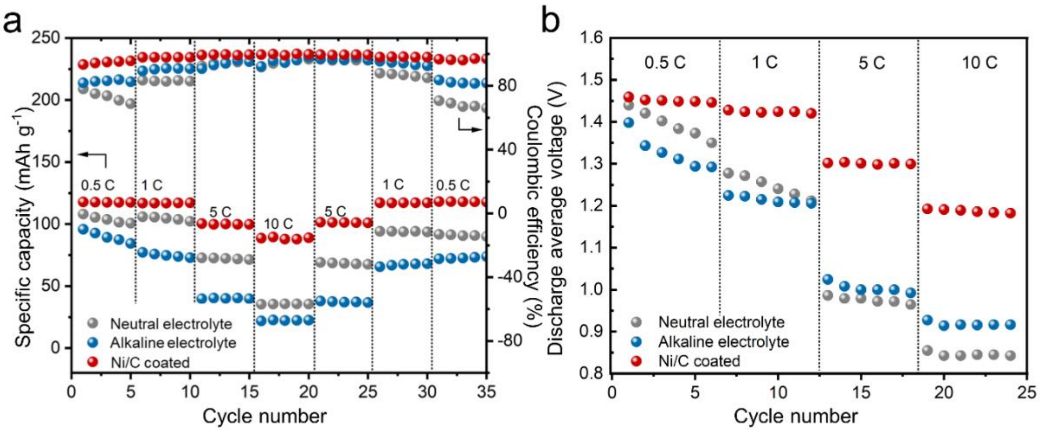

Supplementary Fig. 9 | Electrochemical performance for NMF//NTP full cell. a, Rate capability and corresponding CE for three NMF//NTP cells with, neutral, alkaline and alkaline (with Ni/C coated) electrolytes. **b,** Comparison of average discharge voltage for three cells at varying rates.

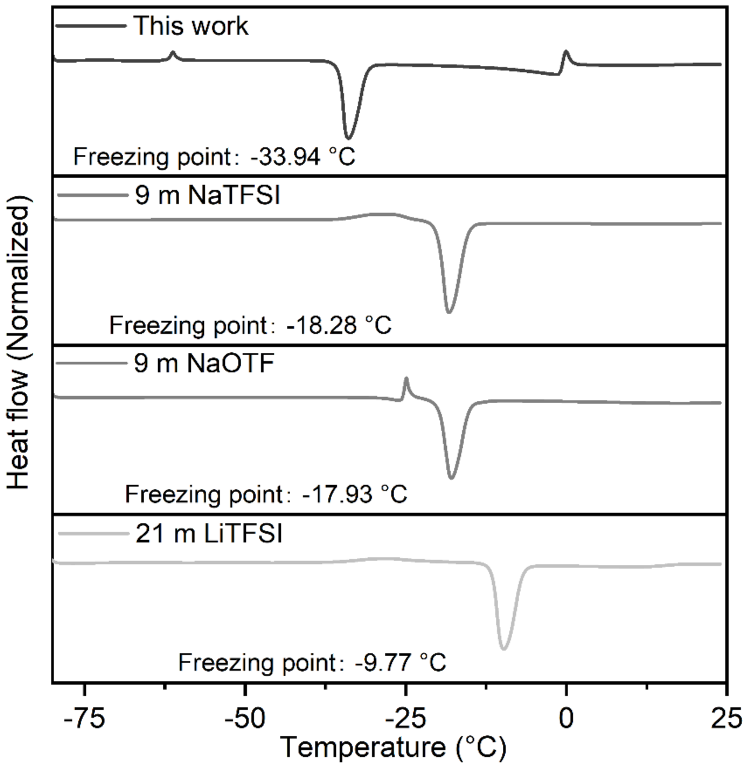

Supplementary Fig. 10 | Differential scanning calorimetry (DSC) for selected electrolytes. Electrolyte in this work exhibits lowest freezing point of −33.94 °C compared with commonly used electrolytes, 9 m NaTFSI, 9 m NaOTF and 21 m LiTFSI.

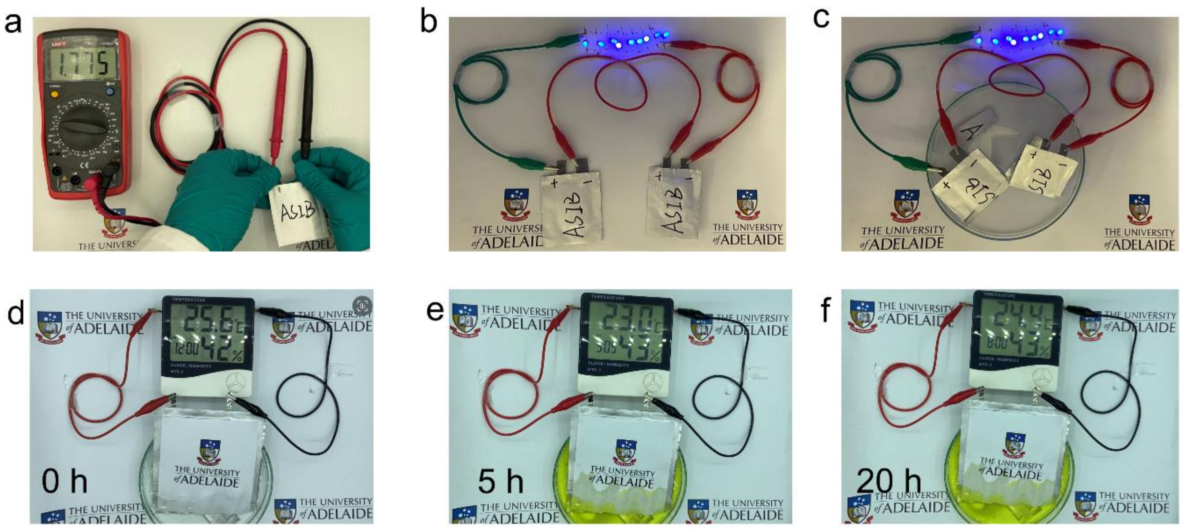

Supplementary Fig. 11 | Testing for safety risks with NMF//NTP pouch cell. a, Output voltage for pouch cell. **b,** Digital photograph of blue-color LED lights powered by two NMF//NTP pouch cells. **c,** Digital photograph of cut pouch cells, powered blue-color lights in water. **d-f,** A cut pouch cell following recharge powering humidity clock in water for > 20 h.

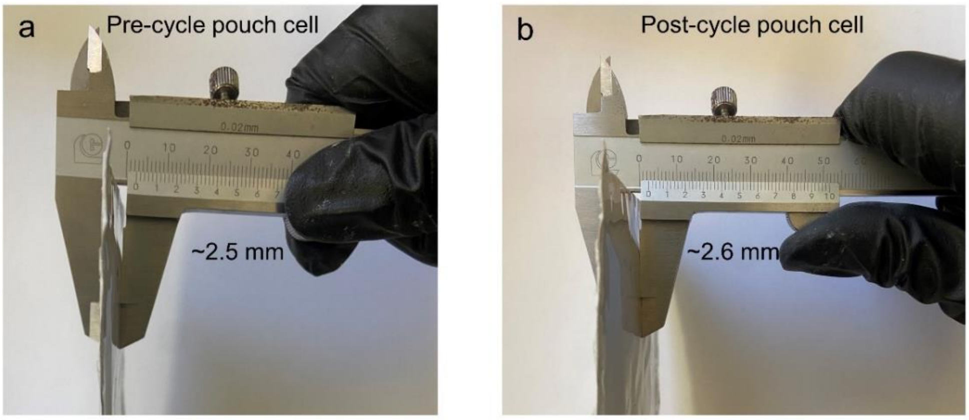

Supplementary Fig. 12 | Digital photograph of pouch cell. Thickness of pouch cell **a,** Before, and **b,** Following cycling**.**

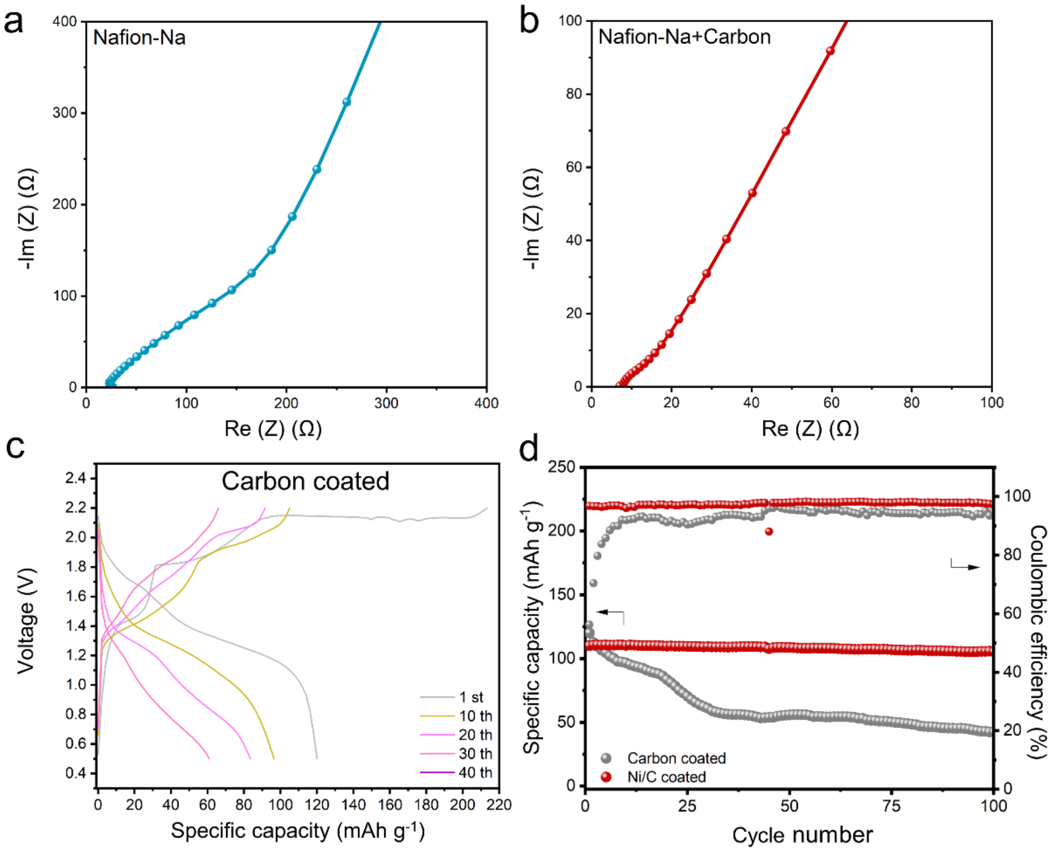

Supplementary Fig. 13 | Function of carbon in coating. EIS spectra for **a,** Nafion-Na and **b,** Nafion-Na and 25 m/m% carbon in Nafion-Na (following addition of 10 μL alkaline electrolyte) using stainless steel//coating//stainless steel cell configuration**. c**, Charge/discharge profiles for NMF//NTP full cell following coating carbon. **d**, Comparison of cycling performance for NMF//NTP full cell following coating carbon with that following coating Ni/C in alkaline electrolyte.

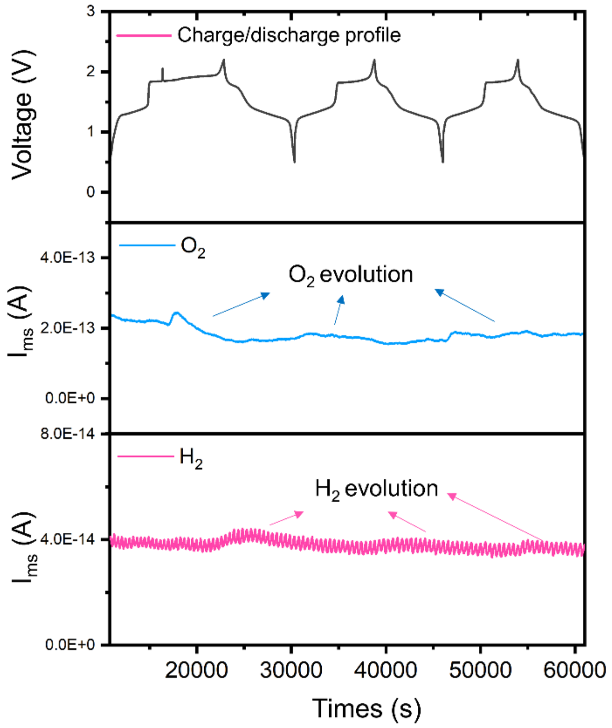

Supplementary Fig. 14 *Operando* DEMS to determine H_2 and O_2 evolution during NMF//NTP battery cycling in neutral electrolyte at 0.5 C (Voltage range 0.5 to 2.2 V).

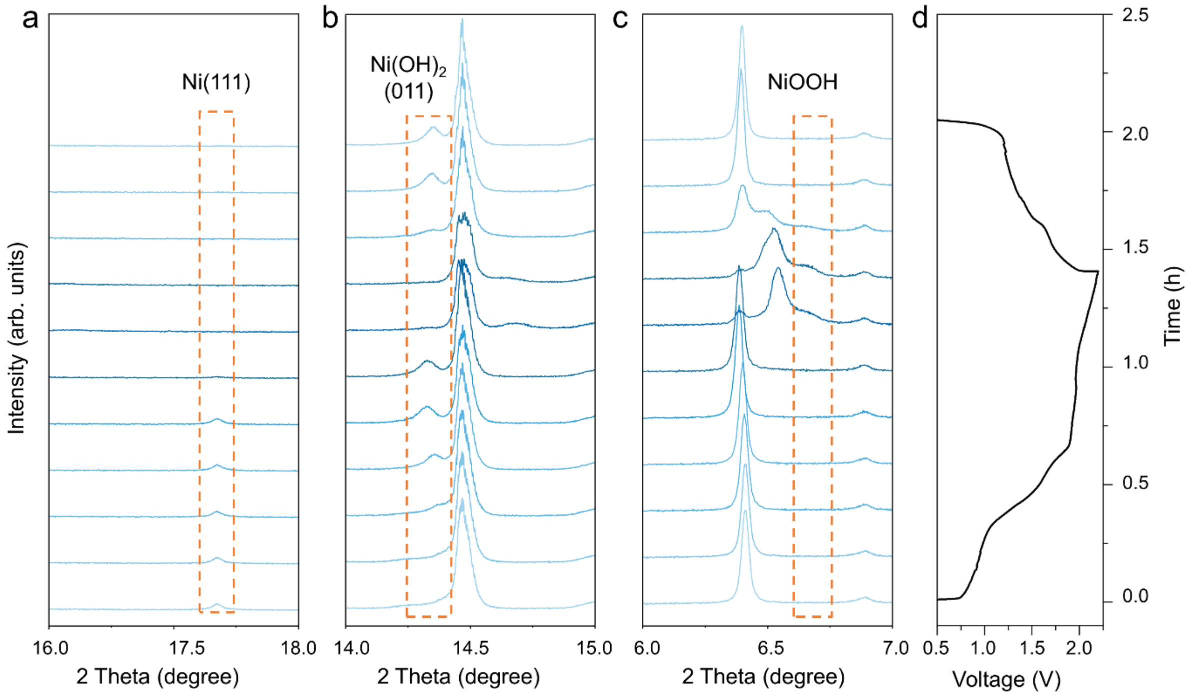

Supplementary Fig. 15 | *Operando* synchrotron XRPD (λ = 0.5903) for NMF//NTP full cells with Ni/C coating in range 0.5 to 2.2 V. a, b and **c,** XRPD patterns for Ni/C coated NMF cathode during 1st charge/discharge and **d,** Corresponding charge/discharge profile (right).

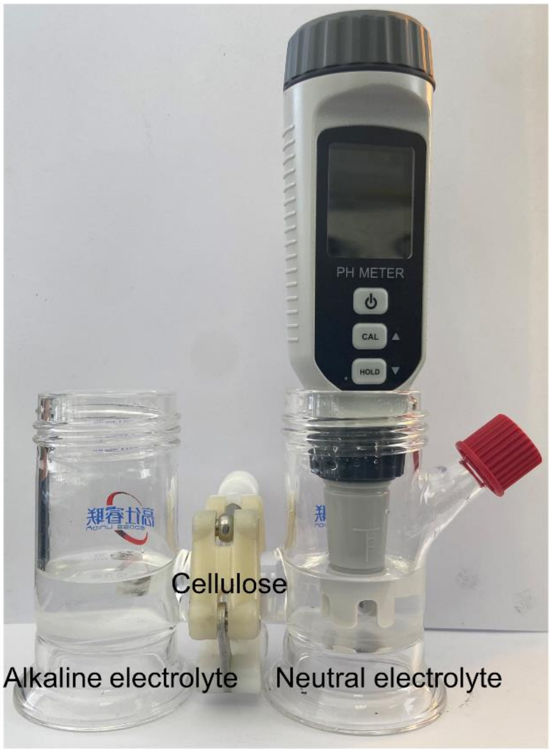

Supplementary Fig. 16 | Digital photograph of the H-cell to test barrier effect of coatings.

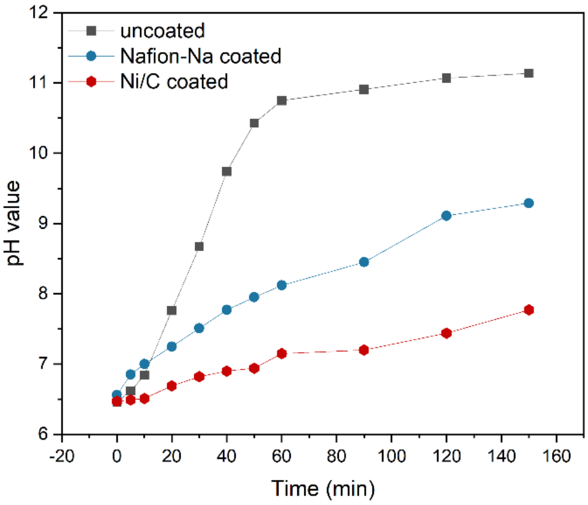

Supplementary Fig. 17 | Increasing pH rate in OH^- penetration tests using H-cells.

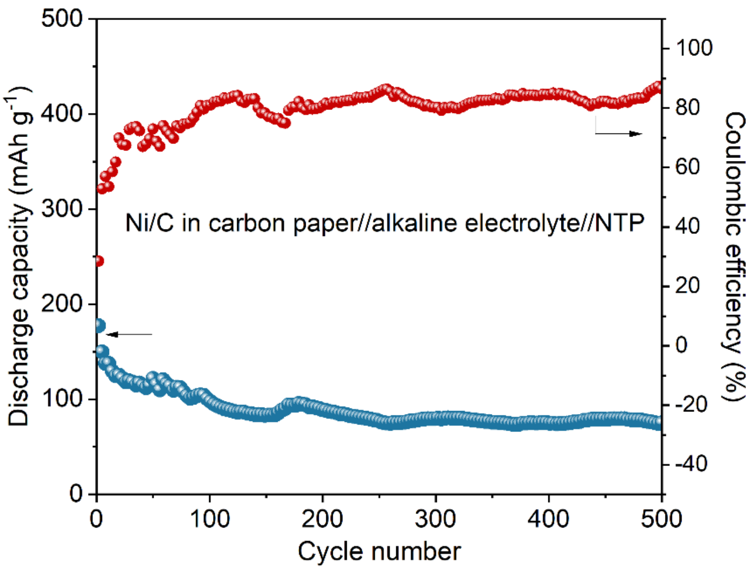

Supplementary Fig. 18 | Cycling performance for Ni/C//alkaline electrolyte//NTP full batteries (pre-cycled for 1 cycle).

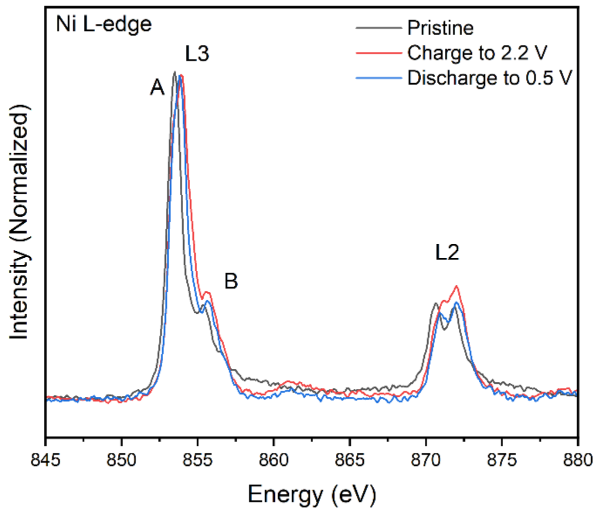

Supplementary Fig. 19 | Soft XAS spectra for Ni/C coated NMF cathode in pristine state, charged to 2.2 V, and discharged to 0.5 V (Ni L-edge).

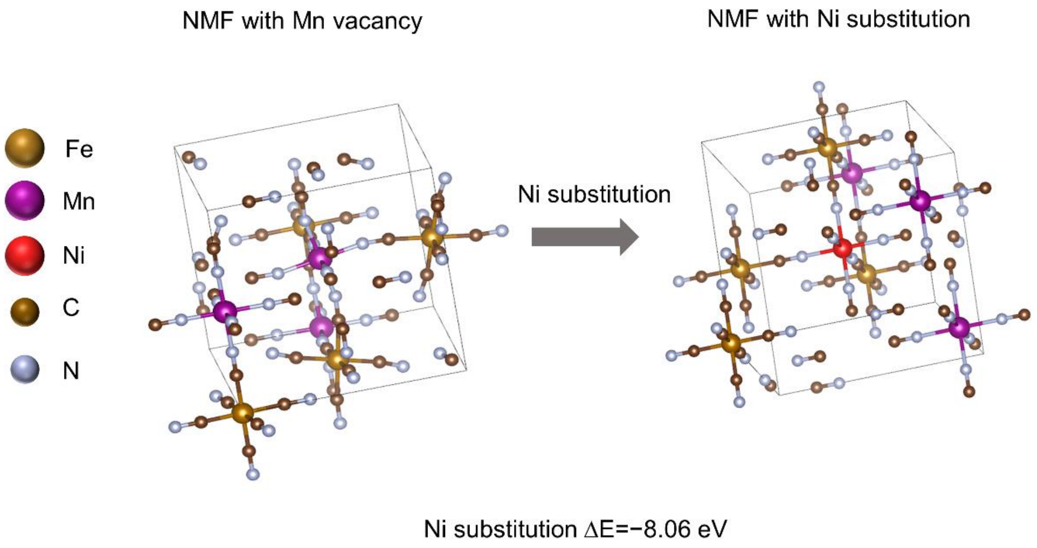

Supplementary Fig. 20 | DFT computations for Ni substitution energy *via* comparing energy of NMF with a Mn vacancy and NMF with Ni substitution.

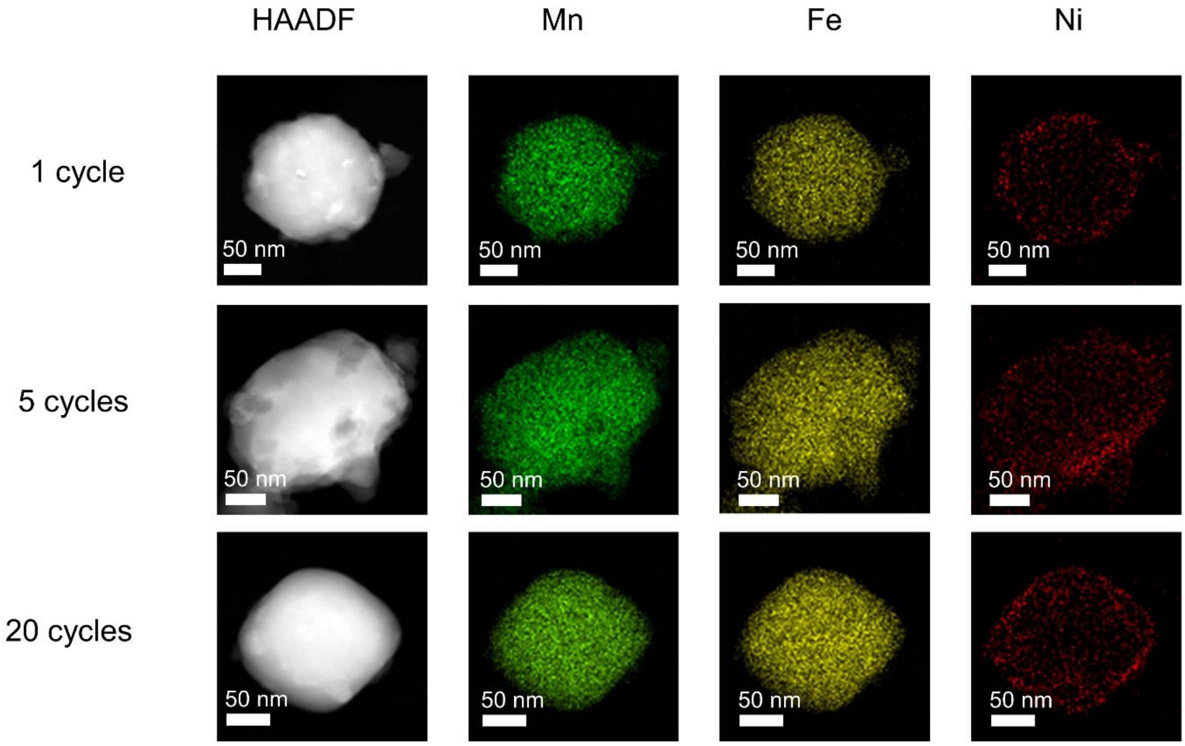

Supplementary Fig. 21 | STEM-EDS mapping for NMF particles with Ni/C coating cycled in alkaline electrolyte following 1st, 5th and 20th cycle.

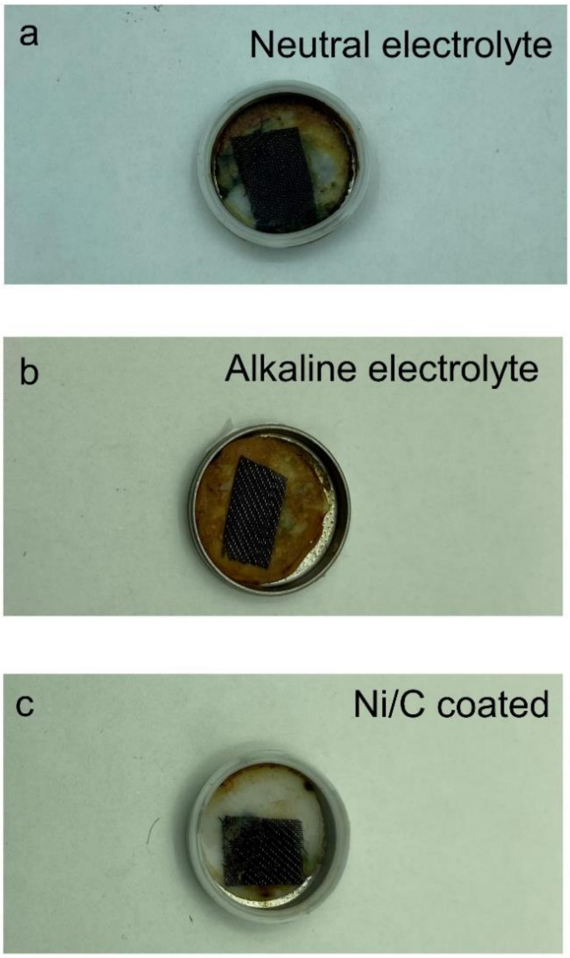

Supplementary Fig. 22 | Digital photographs of cycled batteries. Digital photographs of cycled coin cells in **a,** Neutral **b,** Alkaline and **c,** Alkaline electrolyte with Ni/C coating.

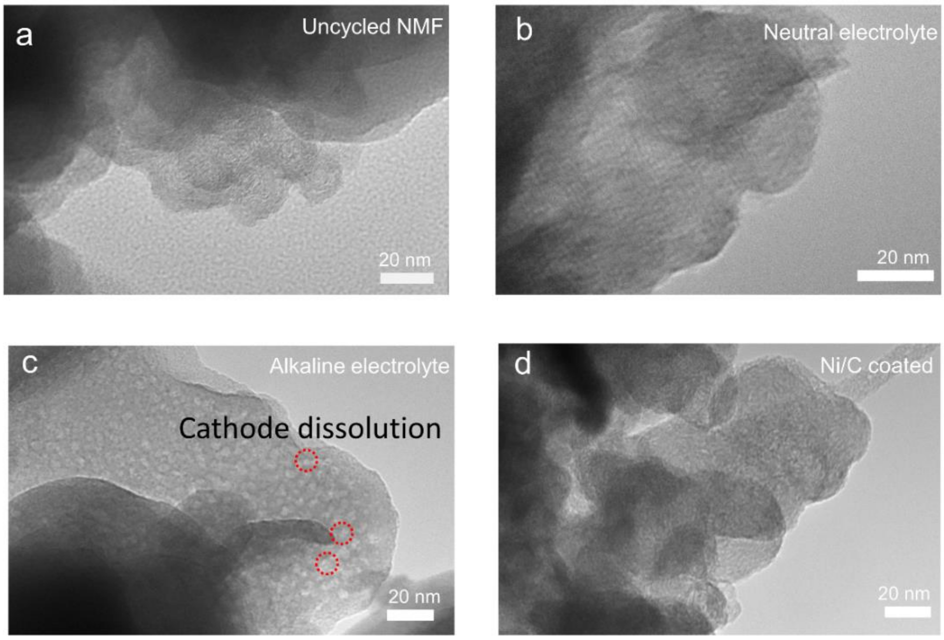

Supplementary Fig. 23 | TEM images of uncycled NMF and cycled NMF. a, Uncycled NMF**. b,** NMF cycled in neutral electrolyte. **c,** NMF cycled in alkaline electrolyte. **d,** Ni/C coated NMF cycled in alkaline electrolyte.

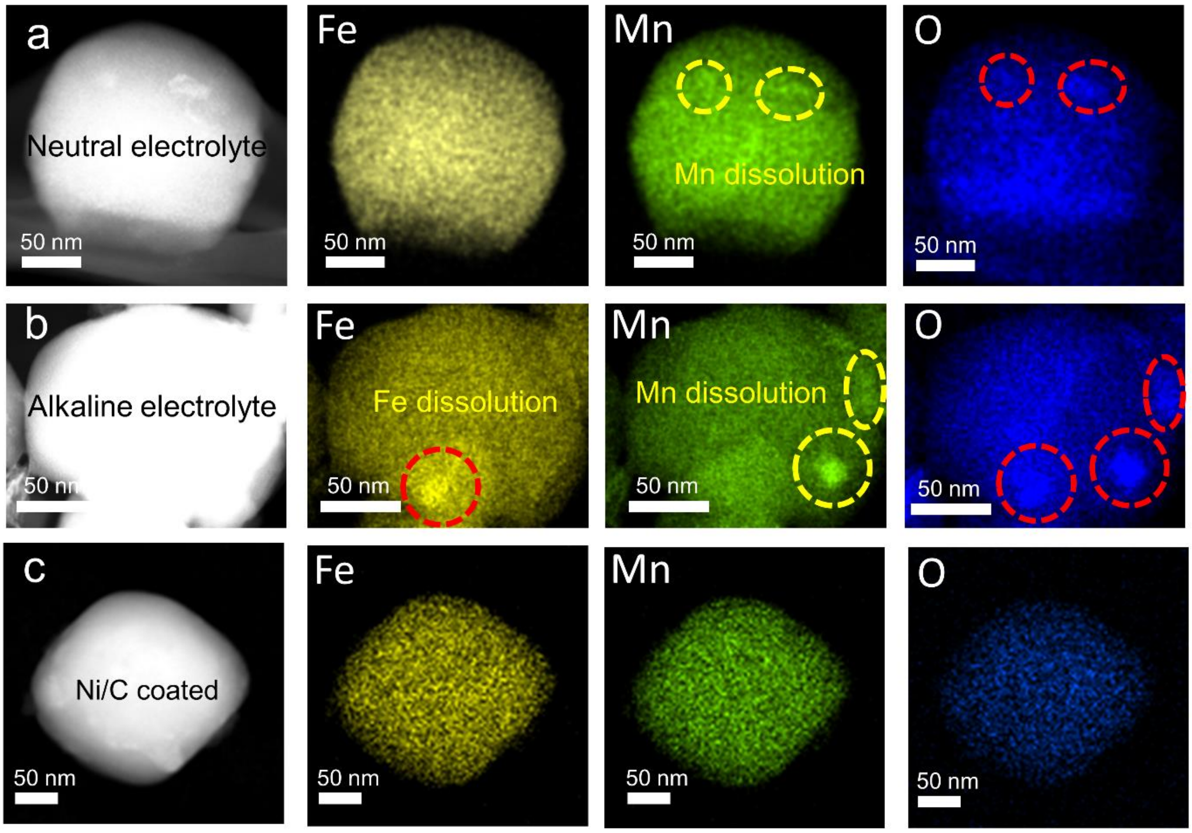

Supplementary Fig. 24 | STEM-EDS mapping for cycled NMF electrode. Electrode cycled in **a**, Neutral electrolyte **b**, Alkaline electrolyte and **c**, Ni/C coating.

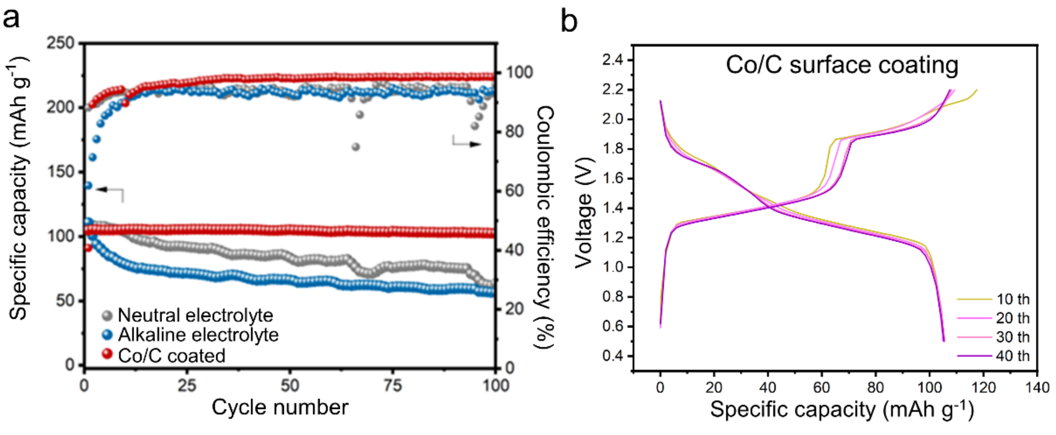

Supplementary Fig. 25 | Cycling performance for NMF//NTP full cells with Co/C coating in alkaline electrolyte. a, Cycling performance for NMF//NTP full cells in selected systems at 1 C with NMF cathode coated with Co/C. **b**, Charge/discharge curves for NMF//NTP cells with NMF cathode coated with Co/C.

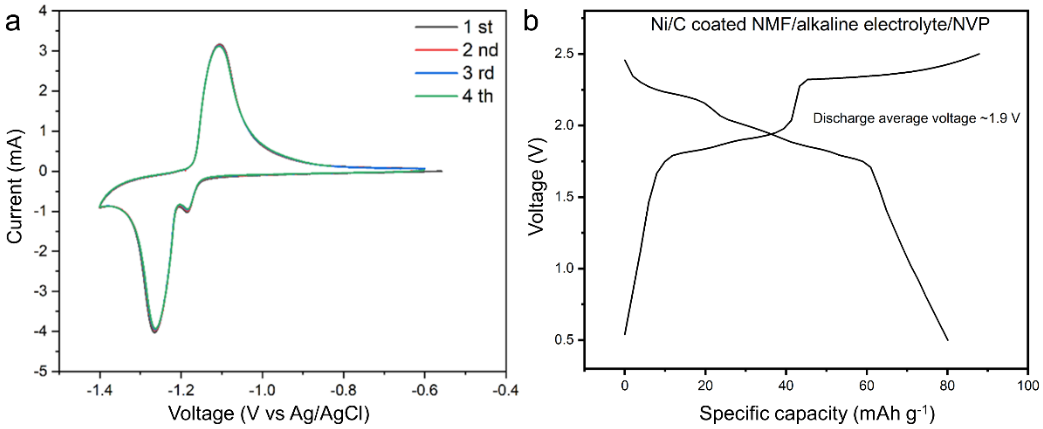

Supplementary Fig. 26 | Electrochemical performance for Ni/C coated NMF/$Na_3V_2(PO_4)_3$@C in alkaline electrolyte. a, CV curves for $Na_3V_2(PO_4)_3$@C using three-electrode with Ag/AgCl with sat. KCl solution as the reference electrode, and carbon black as the counter electrode. **b**, Charge/discharge profiles for $Na_3V_2(PO_4)_3$@C/NMF full cell with Ni/C coating.

Supplementary Tables

Supplementary Table 1 | Viscosity and pH for selected electrolytes.

**Electrolyte**	**pH**	**Viscosity (mPa·s)**
This work	12.3	6.0 at 23.5 °C
21 m LiTFSI (Tested in this work)	~7	46.8 at 23.5 °C
9 m Na CF_3SO_3[4]	~7	4.5 at 25 °C
22 m KCF_3SO_3[4]	~7	6.5 at 25 °C
9 m NaOTF + 22 m TEAOTF[5]	~7	30.2 at 25 °C
8 m LiOAc +32 m KOAc[6]	~9	374 at 25 °C
19.4 m LiTFSI + 8.3 m LiBETI[7]	~7	203 at 30 °C

Supplementary Table 2 | Comparison of lifespan and energy density of this work with reported aqueous Na full batteries.

Battery	Energy density[a] (Wh kg^{-1})	Mean voltage (V)	Cycling life (Capacity retention, Cycles, Rate)
This work	~88.9	1.46	~100 %, 200 cycles, 1 C<br>74.3 %, 13,000 cycles, 10 C
NMF//polymer electrolyte//NTP[13]	86	1.36	80 %, 4,000 cycles, 10 C
NMF//eutectic electrolyte//NTP[14]	~80	1.35	74.5 %, 1,000 cycles, 4.2 C
$Na_{1.88}Mn[Fe(CN)_6]_{0.97}\cdot1.35H_2O$// $NaTiOPO_4$[5]	71	1.74	76 %, 800, 1 C
$Na_3V_2(PO_4)_3$// $Na_3V_2(PO_4)_3$[15]	70	1.75	87.5 %,100,1 C
NaCoHCF//NTP[16]	67	1.33	98 %, 100 cycles, 5 C
$Na_3(VOPO_4)_2F$//NTP[17]	64	1.44	77 %, 500 cycles, 1 C
NMF//17 m $NaClO_4$//NTP[18]	~60	1.3, 1.8	~60 %, 100 cycles, 2 mA cm^{-2}
$Na_2MnFe(CN)_6$//$KMnCr(CN)_6$[19]	58	1.6	75 %, 100 cycles, 30 C
$Na_2Zn_3Fe(CN)_6$//NTP[20]	55	1.6	100 %, 1,000 cycles, 10 C
NaCuHCF//NTP[21]	48	1.4	88 %, 1,000 cycles, 10 C
$Na_2NiFe(CN)_6$//NTP[22]	42.5	1.47	88 %, 100 cycles, 5 C
$Na_3MnTi(PO_4)_3$// $Na_3MnTi(PO_4)_3$[23]	40	1.4	98 %, 250 cycles, 1 C
$K_{0.27}MnO_2$//NTP[24]	34	0.8	80 %, 100 cycles, 1 C
$Na_3V_2O_{2x}(PO_4)_2F_{3-2x}$ //NTP[25]	31	1.47	75 %, 400 cycles, 10 C
$Na_{0.66}Mn_{0.66}Ti_{0.44}O_2$//NTP[26]	31	1	92.7 %, 1,200 cycles, 1 C
$NaMnO_2$//NTP[27]	30	0.81	75 %, 500 cycles, 10 C

[a] Based on the total mass of cathode and anode, the mass of Ni/C has been included in this work.

Supplementary Table 3 | Data for computation of cell-level energy density.

Cell component	Parameter	Type/amount	Unit
Cathode	Active material (AM)	NMF	
	AM capacity	116	mAh g^{-1}
	Coating mass	0.03	g cm^{-2}
	Loading	95.50 %	
	Binder	PTFE[1]	
	Conductive agent	Super P	
	Ni/C coating	1 %	
	Current collector	Ti foil	
	Thickness	6	µm
Anode	Active material (AM)	NTP	
	AM capacity	120	mAh g^{-1}
	coating mass	0.03070845	g cm^{-2}
	Loading	96.50 %	
	Binder	PTFE	
	Conductive agent	Super P	
	Current collector	Ti foil	
	Thickness	6	µm
Electrolyte	Salt	$NaClO_4$	
	Solvent	H_2O	
	Electrolyte/Capacity Ratio	2.5	g Ah
Separator	Celgard		
	Mass	1.09	mg cm^{-2}
	Length	10.6	cm
	Width	10.6	cm
Cell geometry	Size of CE[2]	10*10	cm^2
	Size of AE[3]	10.3*10.3	cm^2
	Size of tabs	1	cm^2
	Size of Separator	10.6*10.6	cm^2
	N/P Ratio	1.07	
	Size of Al–plastic film	12.6*25.2	cm^2
	Cathode Layers	10	
	Anode Layers	11	
	Separator Layers	22	
	Mass of Ni Tabs	0.2167	g cm^{-2}
	Number of Ni Tabs	2	ea
Cell	Output Voltage	1.46	V
	Capacity	6.64	Ah
	Total mass	159	g
	Energy density	61.0	Wh kg^{-1}

[1] PTFE: polytetrafluoroethylene
[2] CE: cathode electrode
[3] AE: anode electrode

Supplementary Table 4 | Comparison of this work with selected commercial batteries.

**Battery**	**Element abundance (ppm)**	**Lifespan (Cycles)**	**Energy density (Wh kg^{-1})**	**Safety**	**Environmental benignity**	**Cost-efficacy**
This work	Fe (41000)<br>Mn (950)<br>Na (23000)<br>Ti (5600)<br>Ni (80)	1,3000	~50	High	High	High
Lead-acid[28]	Pb (14)	300-1,000	25-40	High	Low	High
Ni-MH[29]	Ni (80)<br>La (32)	200-1,200	50-85	Medium	Medium	Medium
Li-ion[30](LFP/graphite)	Li (20)	160-2,000	160	Low	Medium	Medium
Super-capacitor[29]	C (480)	5,000-10^5	5-10	High	High	Low
Flow battery[28] (Vanadium redox)	V (120)	5,000-10^4	10-25	High	Low	Medium

Supplementary Table 5 | Adsorption energy for adsorbed H, OH and H_2O at most stable adsorption sites on (200) and (111) facets of Ni nanoparticles under differing electro-potential.

**Voltage**	**Facet (2 0 0)**			**Facet (1 1 1)**		
**(V *vs.* SHE)**	**$*H_2O$**	***OH**	***H**	**$*H_2O$**	***OH**	***H**
0.0	−0.53	−1.17	0.61	−1.61	−2.52	−4.21
0.5	−0.62	−1.25	0.28	−1.12	−1.81	−0.86
1.0	−0.41	−1.08	0.33	−1.13	−1.72	0.90

Supplementary Table 6 | Elemental atomic fraction based on EDS mapping for NMF following different cycles.

Cathode	Fe (%[a])		Mn (%[a])		Ni (%[a])	
	Atomic fraction	Atomic error	Atomic fraction	Atomic error	Atomic fraction	Atomic error
NMF with Ni/C following cycling in alkaline electrolyte for 1 cycle	44.63	7.64	53.19	6.00	2.18	0.40
NMF with Ni/C following cycling in alkaline electrolyte for 5 cycles	44.70	7.66	53.24	6.01	2.06	0.38
NMF with Ni/C following cycling in alkaline electrolyte for 20 cycles	45.00	7.73	53.05	6.02	1.95	0.36

[a] Total amount of Fe, Mn and Ni was normalized to 100 %.

Supplementary Table 7 | Detailed structural information on samples cycled for 1st cycle following Rietveld refinement.

Sample	a =b=c (Å)	α=β=γ	Volume ($Å^3$)	Size (μm)	R_{wp} (%)
NMF cathode following cycling in alkaline electrolyte	5.26358	90°	145.829	0.0454	5.36
Ni/C coated NMF cathode following cycling in alkaline electrolyte	5.28161	90°	147.333	0.2229	5.35

References

1. Lin, F. *et al.* Surface reconstruction and chemical evolution of stoichiometric layered cathode materials for lithium-ion batteries. *Nat. Commun.* **5**, 3529 (2014).
2. Tian, C. X. *et al.* Depth-dependent redox behavior of $LiNi_{0.6}Mn_{0.2}Co_{0.2}O_2$. *J. Electrochem. Soc.* **165**, A696-A704 (2018).
3. Han, J., Zhang, H., Varzi, A. & Passerini, S. Fluorine-free water-in-salt electrolyte for green and low-cost aqueous sodium-ion batteries. *ChemSusChem* **11**, 3704-3707 (2018).
4. Jiang, L. W. *et al.* Building aqueous K-ion batteries for energy storage. *Nat. Energy* **4**, 495-503 (2019).
5. Jiang, L. W. *et al.* High-voltage aqueous Na-ion battery enabled by inert-cation-assisted water-in-salt electrolyte. *Adv. Mater.* **32**, 1904427 (2020).
6. Lukatskaya, M. R. *et al.* Concentrated mixed cation acetate "water-in-salt" solutions as green and low-cost high voltage electrolytes for aqueous batteries. *Energy Environ. Sci.* **11**, 2876-2883 (2018).
7. Yamada, Y. *et al.* Hydrate-melt electrolytes for high-energy-density aqueous batteries. *Nat. Energy* **1**, 16129 (2016).
8. Xie, J., Liang, Z. J. & Lu, Y. C. Molecular crowding electrolytes for high-voltage aqueous batteries. *Nat. Mater.* **19**, 1006-1011 (2020).
9. Xu, J. J. *et al.* Aqueous electrolyte design for super-stable 2.5 V $LiMn_2O_4||Li_4Ti_5O_{12}$ pouch cells. *Nat. Energy* **7**, 186-193 (2022).
10. Ge, J. M., Fan, L., Rao, A. M., Zhou, J. & Lu, B. G. Surface-substituted Prussian blue analogue cathode for sustainable potassium-ion batteries. *Nat. Sustain.* **5**, 225-234 (2022).
11. Shang, Y. X. *et al.* An "ether-in-water" electrolyte boosts stable interfacial chemistry for aqueous lithium-ion batteries. *Adv. Mater.* **32**, 2004017 (2020).
12. Yang, C. Y. *et al.* Aqueous Li-ion battery enabled by halogen conversion-intercalation chemistry in graphite. *Nature* **569**, 245-250 (2019).
13. Hou, Z. G. *et al.* Bipolar electrode architecture enables high-energy aqueous rechargeable sodium ion battery. *Nano Res.* **15**, 5072-5080 (2022).
14. Liu, T. *et al.* Water-locked eutectic electrolyte enables long-cycling aqueous sodium-ion batteries. *ACS Appl. Mater. Interfaces* **14**, 33041-33051 (2022).
15. Jin, T. *et al.* High-energy aqueous sodium-ion batteries. *Angew. Chem. Int. Ed.* **60**, 11943-11948 (2021).

16. Wu, X. Y. *et al.* Vacancy-free Prussian blue nanocrystals with high capacity and superior cyclability for aqueous sodium-ion batteries. *ChemNanoMat* **1**, 188-193 (2015).
17. Reber, D., Kuhnel, R. S. & Battaglia, C. Suppressing crystallization of water-in-salt electrolytes by asymmetric anions enables low-temperature operation of high-voltage aqueous batteries. *ACS Mater. Lett.* **1**, 44-51 (2019).
18. Nakamoto, K., Sakamoto, R., Ito, M., Kitajou, A. & Okada, S. Effect of concentrated electrolyte on aqueous sodium-ion battery with sodium manganese hexacyanoferrate cathode. *Electrochem.* **85**, 179-185 (2017).
19. Nakamoto, K., Sakamoto, R., Sawada, Y., Ito, M. & Okada, S. Over 2 V aqueous sodium-ion battery with Prussian blue-type electrodes. *Small Methods* **3**, 1800220 (2019).
20. Shao, M. M. *et al.* A high-voltage and cycle stable aqueous rechargeable Na-ion battery based on $Na_2Zn_3[Fe(CN)_6]_2$ $NaTi_2(PO_4)_3$ intercalation chemistry. *ACS Appl. Energy Mater.* **2**, 5809-5815 (2019).
21. Wu, X. Y. *et al.* Energetic aqueous rechargeable sodium-ion battery based on $Na_2CuFe(CN)_6$-$NaTi_2(PO_4)_3$ intercalation chemistry. *ChemSusChem* **7**, 407-411 (2014).
22. Wu, X. Y., Cao, Y. L., Ai, X. P., Qian, J. F. & Yang, H. X. A low-cost and environmentally benign aqueous rechargeable sodium-ion battery based on $NaTi_2(PO_4)_3$-$Na_2NiFe(CN)_6$ intercalation chemistry. *Electrochem. Commun.* **31**, 145-148 (2013).
23. Gao, H. C. & Goodenough, J. B. An aqueous symmetric sodium-ion battery with NASICON-structured $Na_3MnTi(PO_4)_3$. *Angew. Chem. Int. Ed.* **55**, 12768-12772 (2016).
24. Liu, Y. *et al.* Hollow $K_{0.27}MnO_2$ nanospheres as cathode for high-performance aqueous sodium ion batteries. *ACS Appl. Mater. Interfaces* **8**, 14564-14571 (2016).
25. Kumar, P. R., Jung, Y. H., Lim, C. H. & Kim, D. K. $Na_3V_2O_{2x}(PO_4)_2F_{3-2x}$: A stable and high-voltage cathode material for aqueous sodium-ion batteries with high energy density. *J. Mater. Chem. A* **3**, 6271-6275 (2015).
26. Suo, L. M. *et al.* "Water-in-salt" electrolyte makes aqueous sodium-ion battery safe, green, and long-lasting. *Adv. Energy Mater.* **7**, 1701189 (2017).
27. Hou, Z. G., Li, X. N., Liang, J. W., Zhu, Y. C. & Qian, Y. T. An aqueous rechargeable sodium ion battery based on a $NaMnO_2$-$NaTi_2(PO4)_3$ hybrid system for stationary energy storage. *J. Mater. Chem. A* **3**, 1400-1404 (2015).

28. Yang, Z. G. *et al*. Electrochemical energy storage for green grid. *Chem. Rev.* **111**, 3577-3613 (2011).
29. Opiyo, N. Energy storage systems for PV-based communal grids. *J. Energy Storage* **7**, 1-12 (2016).
30. Hesse, H. C., Schimpe, M., Kucevic, D. & Jossen, A. Lithium-ion battery storage for the grid - A review of stationary battery storage system design tailored for applications in modern power grids. *Energies* **10**, 2107 (2017).
