# Supplementary material for: Alkaline-based aqueous sodium-ion batteries for large-scale energy storage
Source: Nat Commun. 2024 Jan 17;15:575. doi: 10.1038/s41467-024-44855-6 (PMC10794691; doi:10.1038/s41467-024-44855-6)
Supplement: Supplementary file 3 — Description of Additional Supplementary Files [file 41467_2024_44855_MOESM3_ESM.pdf]

Description of Additional Supplementary Files

Supplementary Movie 1 The cut pouch cells were immersed in water to power blue lights.

Supplementary Movie 2 The cut pouch cell was immersed in water to power a fan.

Supplementary Movie 3 The cut pouch cell was immersed in water to power a meter.
